# Early Learning From a Low-Resource COVID-Response Virtual Mental Health Crisis Ward: Mixed Methods Study

**DOI:** 10.2196/39861

**Published:** 2022-11-04

**Authors:** Katherine Lee, Shay-Lee Bolton, Ravit Shterenberg, James M Bolton, Jennifer M Hensel

**Affiliations:** 1 Max Rady School of Medicine University of Manitoba Winnipeg, MB Canada; 2 Department of Psychiatry University of Manitoba Winnipeg, MB Canada

**Keywords:** virtual ward, mental health, COVID-19, implementation, driver diagram, virtual care, virtual health care, acceptance, psychiatric support, crisis support, provider perspectives

## Abstract

**Background:**

The COVID-19 pandemic was accompanied by the accelerated uptake of virtual care, leading to a proliferation of virtual ward models as alternatives to facility-based care. Early in the pandemic, our program implemented a virtual mental health crisis ward (vWard) to provide options for individuals requiring intense psychiatric and/or crisis support but who preferred to remain in the community and were deemed safe to do so.

**Objective:**

The aim of this study was to identify early learnings from the vWard, which was implemented rapidly in a resource-constrained environment, to inform the future state should it be sustained beyond the pandemic.

**Methods:**

Mixed methods of data collection were used to evaluate provider perspectives on the vWard, develop archetypes for individuals who are a good fit for the vWard model, and create a driver diagram. Data sources included an anonymous survey of clinical and managerial staff involved in the vWard, a service planning workshop, and program discharge forms for all individuals admitted between March 2020 and April 2021. Survey responses were coded for themes under categories of “benefits” and “challenges.” Discharge forms where the team indicated that the vWard was a good fit for an individual were examined for characteristics common to these admissions. These findings were reviewed in the service planning workshop and refined with input from the participants into patient archetypes. A driver diagram was created for the future state.

**Results:**

Survey respondents (N=60) represented diverse roles in crisis services and the vWard team. Ten providers took part in the service planning workshop. A total of 467 discharge forms were reviewed. The vWard was felt to be a model that worked by 39 survey respondents, one respondent felt it did not work, and the remaining participants had no response. Several benefits for the individual and the system were identified alongside challenges, including certain processes and materials related to the nature of rapid implementation during the pandemic, and others due to lack of fit for certain individuals. The model was felt to be a good fit for 67.5% of admissions. Four patient archetypes representing a good fit with the model were developed. The driver diagram connected the program aim with primary drivers of (1) reduce barriers to care; (2) improve outcomes; and (3) provide collaborative, patient- and family-centered care to secondary drivers and interventions that leveraged virtual technology among other crisis care interventions.

**Conclusions:**

Despite some challenges, the vWard demonstrated high levels of provider acceptance and a range of mechanisms by which the model works for a variety of patient archetypes. These early learnings provide a foundation for growth, sustainability, and spread of this model going forward beyond the pandemic.

## Introduction

The COVID-19 pandemic drastically accelerated the uptake of virtual care [1-3], as many health systems sought ways to continue to provide care in accordance with new public health guidelines and to prevent the spread of SARS-CoV-2. The mental health field was uniquely positioned as a leader in telemedicine, as telemental health had a strong real-world evidence base for its effective use prior to the pandemic [2]. This was in part facilitated by the fact that physical exams are generally not required for mental health visits, whereas this is a frequently cited concern among practitioners in other fields when faced with the transition to telemedicine [3-5]. Therefore, as physical spaces in medical facilities closed or reduced capacity to accommodate physical distancing during the pandemic, mental health care programs around the world, aided by relaxed regulatory constraints, were able to rapidly transition to or expand virtual-based care that supported patients in their homes [2,6].

The pandemic also saw the proliferation of virtual ward models that leveraged technology to reduce the need for hospitalization. Examples of virtual wards specifically designed to assess and manage individuals with COVID-19 infection emerged across the globe, with studies reporting on these models in the Americas, Europe, Australia, and Asia [7]. Virtual ward models are expansions of hospital-at-home models, where a health care team provides treatment to a patient with an acute condition in the patient’s home [8]. A recent systematic review of hospital-at-home programs showed evidence of benefit on patient/family- and system-level outcomes for chronic respiratory and cardiac disease, with greater cost-savings potential in the admission avoidance models compared to the models aimed at accelerating discharge from hospital [8]. Mental health crisis outreach models represent another form of hospital-at-home models in that they target individuals at high risk of hospitalization to offer crisis stabilization through in-person care in the community [9]. Prior to COVID-19, technology-enhanced virtual wards were building momentum but were not widely adopted and were largely serving individuals with chronic medical diseases [7]. While virtual mental health care delivery to individuals in their homes was increasing prior to the pandemic with the use of personal videoconferencing [10-12], there were no examples of virtual wards specifically designed for the management of a mental health crisis that leveraged technology for remote assessment and intervention.

Within weeks of the COVID-19 pandemic reaching Winnipeg, Manitoba, Canada, our center rapidly opened two virtual mental health crisis units aimed at avoiding admission to the corresponding facilities: one focusing on high-acuity psychiatric presentations to avoid admission to hospital, and the other being a lower-acuity unit to replace several beds on the Crisis Stabilization Unit (CSU) that were closed to adhere to public health guidelines for social distancing. The CSU is a community-based voluntary short-stay facility, staffed by a multidisciplinary team that acts as an alternative to hospitalization for individuals experiencing a mental health crisis [13]. The virtual units (collectively referred to as the virtual ward [vWard]) were designed to deliver all care remotely by email, text messaging, telephone, and/or videoconferencing to patients who were deemed suitable to remain in their homes.

We here report on the early learnings from the rapid implementation and delivery of these collective models in a low-resource environment. We examined the strengths and challenges of the model from the perspectives of the health care teams and the profiles of patients who were felt to be the best fit for the model. Based on these findings, we further developed a driver diagram for the future vision of a comprehensive virtual mental health crisis ward that can act as an alternative to hospital- and facility-based care in the post-COVID era, where the objective is no longer limited to keeping individuals in their homes for public health reasons. Going forward, understanding the mechanisms by which the virtual crisis ward model can be effective and for whom will inform the future of home-based virtual mental health crisis care.

## Methods

### Study Design

This study employed a mixed methods approach to data collection and an integrated analysis to achieve the objectives. Data sources included a voluntary online survey of providers, a service planning workshop, and discharge forms completed on all patients who accessed the virtual ward programs between March 2020 and April 2021.

### Ethics Approval

Research ethics approval for this study was obtained from the University of Manitoba (HS23878 [H2020:196]).

### Setting

The study was based in Winnipeg, the capital city of Manitoba, Canada. Winnipeg has a population of ~780,000, which is served by 3 emergency departments (EDs) and 3 urgent care centers offering 24/7 service. Adults can access mental health crisis care in Winnipeg via these centers or the centrally located Crisis Response Centre (CRC). The CRC is a stand-alone 24/7 walk-in mental health center, which also offers a telephone crisis line, postcrisis follow-up services, and is linked with the CSU. The virtual wards were housed at the CRC and CSU. Eligible individuals were assessed at the CRC, EDs, or urgent care centers in the city, and were deemed safe to remain in the community. This required that the individual was not at imminent risk of harm to themselves or others, participated actively in safety planning, and, when possible, had a secondary contact who would be involved in their care. The higher-acuity vWard required that individuals were assessed by the psychiatry team at the referring sites prior to referral. There were no restrictions on diagnosis or concomitant care.

Referrals were submitted to the vWard teams and patients were contacted the next day for an initial consultation. Initial contact was made by phone or email to arrange a detailed assessment, which was conducted via videoconferencing or phone when video was not available. At this assessment, a plan was made for the individual’s stay, including treatment goals, safety planning, and program offerings. Families and other supports were often involved in these meetings. The higher-acuity beds were managed by a rotation of physician assistants and psychiatrists working at the CRC who offered assessment up to multiple times a day as needed, including diagnostic assessment, risk monitoring, medication management, and supportive care. This team was usually also responsible for providing care at the CRC for individuals presenting on site in crisis. The lower-acuity vWard was managed by a dedicated crisis clinician 7 days a week, usually having training in nursing or social work. The crisis clinician provided 1:1 daily virtual crisis assessment and support, along with optional group classes teaching skills derived from cognitive behavioral therapy and dialectical behavioral therapy. Psychiatric support was available to the lower-acuity beds as required. All patients had access to the 24/7 crisis phone line at the CRC for after-hours support. Medication management, when applicable, was coordinated with the individual’s community pharmacy. vWard admissions were documented in the same electronic patient record used by the CRC, facilitating common access to clinical care details. The target length of stay for the virtual beds was 3-5 days. On average, there were 2 higher-acuity beds and 6 lower-acuity beds available at a given time.

Although the virtual wards operated independently of one another, we have examined them collectively with the natural progression being the merging of the units with shared infrastructure and management to provide a more collaborative, full-spectrum model of care with a dedicated team.

### Data Collection

#### Provider Survey

A voluntary, anonymous open online survey was created using SurveyMonkey and distributed to all clinical and managerial staff who were involved with the vWard anywhere from the point of referral through discharge. An invitation to complete the survey was sent out through the email listservs for the involved services by the principal investigator of the study and forwarded by service leads who encouraged participation. The survey was open for a period of approximately 2 weeks (mid-June to early July 2021). There was no monetary incentive to participate. The survey items were developed by the principal investigator with input from the research team. Respondents were asked to identify their roles, indicate whether they felt that the vWard worked as a model of care (yes/no), and answer a series of free-response questions that included: (1) Who does the virtual unit serve? Describe the patient population. (2) What does the virtual unit do? (3) Why does it work? (4) Why doesn’t it work? (5) What impact of the virtual unit do you perceive for the health care providers?

#### Patient Discharge Forms

Each individual admitted to the vWard had a discharge form completed by a clinical team member at the time of discharge. The discharge forms captured the key elements of the individual’s condition and care delivered during the virtual admission. At the end of the form, the team member was asked to rate if the vWard was a good fit for the individual on a 5-point Likert scale (“strongly disagree” to “strongly agree”), and comment on specific successes and challenges in free text.

#### Service Planning Workshop

An invitation to participate in a virtual service planning workshop was emailed to the same recipients of the provider survey. Survey completion was not required to participate. Workshop recruitment favored a diversity of roles within crisis services to gain a breadth of perspectives. The workshop aimed to expand on findings from the survey and discharge forms by presenting some results for discussion. Participants were engaged in several rounds of feedback as well as a series of planned exercises drawn from quality improvement toolkits (eg, driver diagram, generating change ideas, impact vs feasibility matrix). The main objectives of the workshop were (1) to refine profiles of patients who were felt to be best suited to this model of care and (2) to create a driver diagram for a future virtual crisis ward building on the identified benefits and mechanisms from the survey. In addition to participating in the 2-hour workshop, participants were required to do some preparatory work and participate in some postworkshop follow-up totaling approximately another 2 hours. Compensation was provided in the form of a Can $200 (approximately US $140) electronic gift card to a retailer of their choosing. The 2-hour workshop was held over Zoom, facilitated by a psychiatrist and a medical student who presented the survey results, engaged deeper discussion of those results to develop the impacts and mechanisms of the vWard model for specific patient profiles, and led the participants through the planned exercises. The facilitators were also active participants in the exercises. The workshop was recorded, and all whiteboards, PowerPoint slides, and chat content were saved.

### Data Analysis

The roles and involvement of survey and workshop participants were summarized descriptively. All survey respondents were included regardless of whether every question was answered. Qualitative survey responses were reviewed by 2 study team members and coded for themes pertaining to “benefits” and “challenges” of the vWard. We reviewed responses to all questions and extracted the responses that specifically addressed a benefit or challenge of the intervention. We then used a qualitative content analysis approach [14] to code individual responses and group them into thematic categories. All discharge forms where the fit rating was “agree” or “strongly agree” were selected and reviewed for demographics, clinical presentation, and the free-text comments from the team to cluster into profiles that shared common features. The preliminary profiles were presented to the workshop participants for validation, and feedback was incorporated to further develop them. In addition, a list of individual and clinical features that were generally felt to fit well with the vWard were developed. A driver diagram was drafted during the workshop, which the investigators then further developed with reference to survey responses and workshop participant input. A complete draft was circulated back to the workshop participants for additional feedback prior to finalizing.

## Results

### Survey and Workshop Respondents

In total, there were 60 survey responses, including those in a role of decision-maker/manager/leadership, psychiatrist/physician assistant, crisis unit clinicians, CRC clinical staff, and other/unspecified. There were 10 participants in the provider workshop (Table 1).

**Table 1:** Roles of survey and workshop participants.

Virtual ward role	Survey participants, n (%)	Workshop participants, n (%)
Decision-maker/manager/leadership	10 (16)	1 (10)
Psychiatrist/physician assistant	8 (13)	2 (20)
CSU^a^ clinical staff	16 (26)	3 (30)
CRC^b^ clinical staff	17 (28)	2 (20)
Peer support^c^	0 (0)	2 (20)
Other/unspecified	9 (15)	0 (0)
Total	60 (100)	10 (100)

^a^CSU: Crisis Stabilization Unit.

^b^CRC: Crisis Response Centre.

^c^Peer support did not receive the survey invitation; this role was not formally involved in the virtual units at the time of the study.

### Discharge Forms

Discharge forms were reviewed for 335 admissions to the lower-acuity unit and for 132 admissions to the higher-acuity psychiatric unit. Responses to the statement “Virtual care was a good fit for this patient” were missing for 7 low-acuity and 1 high-acuity admissions. In the remaining admissions, staff agreed or strongly agreed that the lower-acuity unit was a good fit in 214/328 cases (65.2%) and the higher-acuity unit was a good fit in 96/131 cases (73.3%).

### Provider Perspectives: Benefits and Challenges

Survey respondents mostly stated that the vWard worked as a model of care (39/60 responded yes, 1 responded no, and the remaining 20 did not provide a response). Provider perspectives on the benefits of the vWard fell into five thematic categories: (1) provides support to stabilize acute crisis, (2) allows patients to stay in their homes, (3) increases options for patients and care providers, (4) acts as an entry point into the mental health system, and (5) can have better outcomes compared to usual care (Table 2). Providers identified the vWard’s immediate and daily check-ins (for support, monitoring, and early detection of deterioration) and ease of medication support as key factors that facilitated stabilization of acute mental health crises. Providers stated that allowing patients to remain in their homes acted to reduce some of the common patient barriers to typical care, with stated examples including caregiving/work responsibilities, physical distance from the care site, disabilities, and stigma. Furthermore, allowing patients to remain in their homes had the added benefits of reducing overall hospitalizations; maximizing inpatient beds for other users; respecting patient choice; and avoiding patient-oriented risks of inpatient care, such as communicable diseases, violence, and trauma. Providers identified that having vWards available increased the number of options and flexibility of care that they could recommend to patients by offering increased hours in which appointments could occur, different types of care options, and various communication modalities (phone, videoconferencing, virtual resources). Providers also liked having the option to work remotely within the vWard. Regardless of whether the structure of the vWard met the needs of a given patient, providers also identified the benefit of having communication with patients to facilitate referrals to community supports, follow-up care, and/or inpatient care as needed. Lastly, providers identified several potential ways that vWards could provide better outcomes for patients compared to usual care. These included allowing provider assessment of the patient’s function in their usual environment, encouraging practice of coping strategies in real-life situations, facilitating family involvement in care, and creating a smoother transition to the community following discharge.

Despite survey respondents agreeing with the vWard as a model of care, participants outlined several challenges with the existing program. These fell into 4 categories: (1) staff and resource limitations, (2) need for process refinement, (3) limitations of the virtual model compared to usual care, and (4) potential lack of fit for certain individuals (Table 2). The staff and resource limitations outlined by providers included concerns about redeployed and insufficient staff, inadequate equipment/resources due to a limited budget, as well as frustrating technical issues and a steep learning curve associated with the rapid pivot to virtual-based care. The category of “limitations of virtual model compared to usual care” included responses where the virtual model was contrasted with usual inpatient care in terms of assessment/diagnostic accuracy, patient-provider rapport, ability to observe patient behavior, and access to interprofessional supports (eg, social work, nursing).

Providers identified several changes they felt were needed in the administration of the vWard process, including having scheduled appointments to reduce the administrative burden on the provider, and having a standardized protocol for admission, care delivery, and discharge. Providers also felt that the length of stay should be extended for patients needing longer periods to stabilize their crises. Provider responses captured several types of individuals that the vWard model may not be a good fit for. Referred individuals must be self-motivated and actively engaged to see improvements. The virtual aspect of care may be “too convenient” in some cases and lead to disengagement. Patients may also choose virtual care despite a clinical recommendation for inpatient care due to other factors such as social anxiety. Finally, providers highlighted clinician presentations where treatment is more suited to a supervised/closed environment, such as addictions, active suicidal intent/other safety concerns, mania and psychosis, and crises that are a result of the individual’s environment (eg, intimate partner violence, problematic relationships). Additionally, the issue of access to virtual-enabled devices (ie, telephone, internet) was identified.

**Table 2:** Provider perspectives on virtual crisis wards with representative quotes.

Perspectives		Description	Supporting quotes
**Benefits**			
	Provides support to stabilize acute crisis	Immediate and daily check-ins for support and monitoring Daily assessment promotes early detection of deterioration Regular medication support (reminders, adjustments)	“With daily monitoring it serves as an access point to clients who may require further support if their current mental health further deteriorates”
	Allows patients to stay in their homes	Respects patient preference Reduces barriers to typical care (eg, caregiving or work-related responsibilities, physical distance, disabilities, stigma) Avoids patient-oriented risks of inpatient care (communicable diseases, violence, trauma) Reduces hospitalizations and frees up inpatient beds	“This provides individuals with the opportunity to continue with their daily activities and/or remain in their personal environment and still attain support”
	Potential for better outcomes compared to usual care	More flexible care (hours, types of care) Integrates technology (phone, videoconferencing, virtual resources) Allows providers to work remotely	“It allows providers to see patients in their home environment and make sustainable treatment plans”
	Entry point into the mental health system	Facilitates referral to community supports and follow-up care Seamless transition to inpatient care if needed	“The opportunity to bring a client into the in-person unit if they aren’t doing well is a further advantage…”
**Challenges and limitations**			
	Staff and resource limitations	Additional responsibilities contribute to staff burnout Learning curve for providers to pivot to virtual care Technical issues can be frustrating Lack of adequate staffing leads to limited capacity and increased wait times Lack of adequate equipment due to limited budget	“Adding it on to an already very busy service can overwhelm health care providers and contribute to burnout/resentment of the work. These services would likely benefit from their own dedicated team” “Having a ‘waitlist’ defeats the purpose of access to virtual care to those in community requiring supports”
	Processes need refinement	Standardized protocols for admission, care delivery, and discharge Optimization of strategies required for scheduling appointments Maximum length of stay should be extended Risk of shifting individuals who require inpatient care being shifted to virtual due to bed shortages	“A concise procedure/process in writing regarding what to do if there is no contact with a client; how long/how many attempts [to] make” “Reevaluating the length of stay—considering longer”
	Limitations of virtual model compared to usual care	Challenges with virtual assessment accuracy and rapport Lacks observation level of inpatient care Patient must be self-motivated and engaged with care (ie, can be difficult to connect) Lack of typical inpatient interprofessional supports (eg, social work, nursing)	“It’s easy for someone to ‘tune-in’ via Zoom, but also ‘tune out.’ Virtual lacks accountability that one would have with in-person stay” “Sometimes you are stuck trying to manage a very complex case without any of the actual supports you would’ve gotten in the in-person setting”
	Lack of fit for certain individuals	Certain mental health presentations may require a supervised environment and/or closer observation Addictions (sober environments) Active suicidal intent/other safety concerns Mania and psychosis Crises that are a result of the environment (eg, relationship issues) Patients who clinically require inpatient care may opt for virtual (eg, due to social anxiety) Lack of access to resources needed for virtual care Those without access to phone or internet	“[Virtual ward] does not work for patients whose acute crisis presentation had to do with their environment–you can’t always send the patient who [overdose]’d after an argument with tumultuous partner right back to that environment and call them the next day” “Unfortunately, the use of virtual services and the requirement of technology excludes a significant portion of our client population, including those who have lower [socioeconomic status] or experience homelessness”

### Patient Profiles and General “Good” Fit Factors

**Figure 1:**
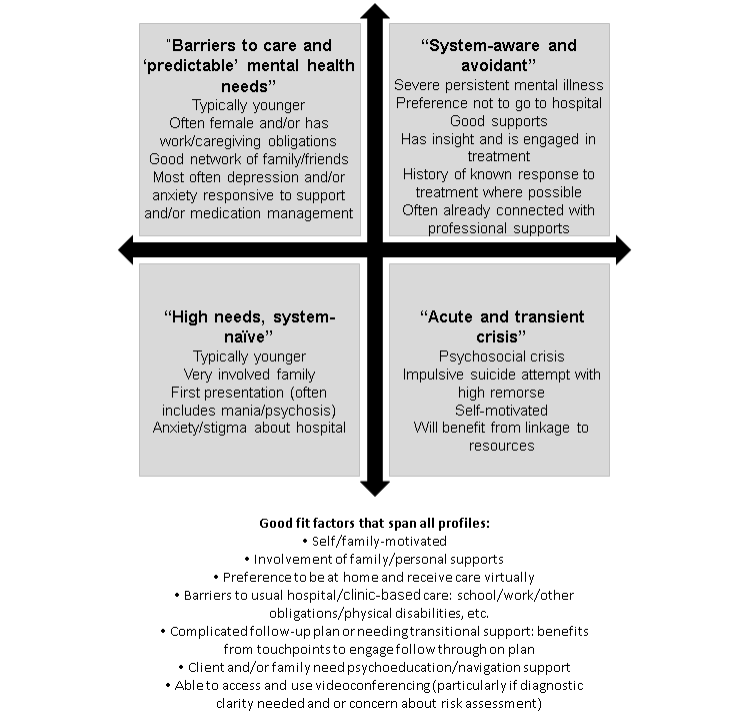
Profiles of patients who were a good fit for the virtual ward (vWard) model.

Individuals who were felt to be a good fit for the vWard fell into four profiles (Figure 1): (1) barriers to care and “predictable” mental health needs; (2) acute and transient crisis; (3) system-aware and avoidant; and (4) high needs, system-naïve. The first profile (barriers to care and “predictable” mental health needs) represents an individual who typically has a common mental disorder likely to be responsive to treatment with medication and/or supportive intervention (ie, depression or anxiety), who has a strong support network and/or is highly reliable to engage and follow through, and who has barriers to seeking traditional hospital- or clinic-based care. A high proportion of this group were females in the postpartum year or with young children. Other barriers to typical care pathways included work and physical disabilities.

The “acute and transient crisis” group is one with a specific event or interpersonal difficulty precipitating the crisis admission that was amenable to brief intervention. This ranged from events such as a relationship breakup to an impulsive suicide attempt with high remorse. This group was motivated to move forward and was open to support and assistance with navigating resources for the longer term.

The “system-aware and avoidant” and “high needs, system-naïve” groups represent individuals who usually have more severe mental health presentations, including severe depression, personality disorders, bipolar, and psychotic disorders. The system-aware and avoidant group was very familiar with the system, often with prior hospitalizations, but preferred not to go to hospital. They were often connected with community resources, including psychiatrists and other mental health professionals, and if experiencing a recurrence of a preexisting problem, had some insight into what the trigger was and/or what would work to improve the symptoms based on prior experience. Conversely, the system-naïve group had little to no prior contact with mental health services; were experiencing a new-onset, usually severe problem; and were very opposed to the idea of hospitalization. These latter two profiles often necessitated the involvement of community-based supports such as family. In a significant number of cases, these presentations did lead to hospitalization, but these transitions were smoother and more acceptable to the individuals and the families who benefitted from the time to attempt recovery at home, receive more education, and collaboratively make the decision that hospitalization was needed.

A list of general “good” fit factors that crossed all patient profiles regarding vWard fit was also created (Figure 1). These included patient factors such as preference for virtual and home-based care, ability to use videoconferencing, self-motivated, involvement of family/personal supports, and the presence of barriers to seeking usual hospital-based care (eg, school or work obligations, mobility challenges). Additionally, the vWards were beneficial to patients and/or families requiring increased support, whether due to a complicated follow-up plan that required points of engagement to ensure follow-through or an increased need for psychoeducation/navigation.

### Driver Diagram

A driver diagram (Multimedia Appendix 1) for the vWard program was developed that included an overall aim (aspiration and scope of the effort), primary drivers (key system components that contribute directly to accomplishing the system aim), secondary drivers (components that contribute to achieving the primary drivers), and interventions (action ideas that can be implemented with the purpose of achieving the aim, with relationship to the drivers). Provider workshop participants identified the overall aim of the vWard:

To deliver an alternative, home-based, collaborative model for acute mental health crisis care that works flexibly with patients and families to reduce barriers and improve outcomes.

The primary drivers that contributed to the aim were identified as (1) reduce barriers to care; (2) improve outcomes; and (3) provide collaborative, patient- and family-centered care. The primary driver of “reduce barriers to care” included both the secondary drivers of system factors such as total system capacity and wait times, and patient “convenience and privacy” factors such as home-based, flexible, personalized, and low-stigma care options. These factors leveraged virtual technology for interventions in communication, direct care delivery, and other program components such as psychotherapy and recreational programming. The primary driver of “improve outcomes” linked to the secondary drivers of promotion of self-management techniques/in vivo stabilization (ie, stabilization in the home environment), stabilization of mental health crises, and seamless transition to other services such as follow-up and in-patient care. The transitions are further aided by providing informational continuity to patients, family, and other care providers. Finally, the primary driver of “collaborative, patient- and family-centered care” links to the secondary drivers of involving a multidisciplinary team and family members/social supports in patient care, as well as performing a patient- and family-centered needs assessment.

## Discussion

### Principal Results

In this paper, we report on our learning from the rapid implementation of vWards for individuals in mental health crises as alternatives for admission to in-person facilities. These models were low-resource, created in response to the public health restrictions imposed by the pandemic, and can provide the foundation to plan for more comprehensive models based on early success and evidence of feasibility. Providers from diverse roles who were involved with care delivery overwhelmingly perceived these to be models that worked and provided benefit to the patient and system. These benefits included patient choice, reduction of barriers to care, improved transitions to other services, and avoidance of risks of in-patient care such as violence or communicable disease. On a system level, the benefits included increased system capacity, improved access, and potential cost-savings. Limitations noted reflected the rapid nature of the implementation with limited resources and a sudden transition to virtual care delivery, in addition to limitations inherent to virtual acute care delivery. Distinct and diverse patient profiles that could particularly benefit from the virtual model were developed. Together, these findings allowed the development of a driver diagram for a comprehensive model that could be delivered in a higher-resource setting beyond the pandemic driven by patient choice, outcomes, and optimization of resources.

### Comparison With Prior Work

Not surprisingly, many of the benefits and mechanisms of impact that were identified mirror those reported in the implementation of community-based crisis resolution teams [9]. Some additional advantages of the virtual model included the ability to reach patients in settings beyond the home to include workplaces. However, the availability of community outreach options was noted as a desired intervention for a future model, recognizing the need to connect more directly with individuals in person whose condition is deteriorating or who are unreachable. To address this, virtual models could partner with police-involved crisis outreach teams [15] as a way to increase the spectrum of care that could be offered and to provide additional comfort for the team when managing more acute presentations. Furthermore, many of the positive effects of the vWard align with those reported in the home-based telemental health literature. The general benefits included decreased barriers to treatment (eg, stigma, social anxiety, physical disabilities), patient convenience, safer environment for providers and patients at risk of violence/behavioral issues, reduced disease transmission, and the option of remote work for providers [4,5,12,16]. It has been noted that the patient-centered approach of telemental health (ie, focusing on removal of barriers and patient convenience) can lead to improved treatment adherence as patients are more satisfied with their care [10,12]. Improved treatment adherence is one of the possible mechanisms by which the vWard could result in improved mental health outcomes compared to usual care.

While providing low-barrier, flexible options for care is beneficial, there were also situations where the vWard was not felt to be appropriate or introduced limitations to proper assessment and management of certain individuals. The model relies on patients being self-motivated and engaged with their care, as there are more distractions in the home environment that could impede focus on self-improvement. If a patient missed virtual appointments, there were few options to contact them for follow-up. Home disturbances and interfering factors have been previously described in the virtual care literature [17] and for mental health care specifically [11], and require attention if care outcomes are to be optimized. Patients who require contained environments, such as those with highly agitated and disorganized presentations, or those needing sober environments will also not be good candidates for vWard admission. Zimmerman et al [18] reported on the virtual transformation of a partial hospitalization program during the pandemic, demonstrating feasibility and retention compared to a historical comparison cohort; however, the users of the virtual model did have lower levels of psychosis, and presence of a primary substance use disorder was an exclusion criterion. Access to virtual care is also a limitation as there is a risk to expose inequities [19]. While the majority of Canadians do have access to virtual technology, a portion do not, and this is often correlated with other measures of marginalization and poor health [19]. This is an area that requires more attention at a population level to ensure equitable access for all.

Many of these factors were also identified in our patient profile based on good fit. The generally “good” factors capture the presence of barriers and preference, ability, and motivation for the virtual care model compared with in-person care. The specific profiles that were elucidated from the data exemplify the diversity of individuals who can be managed in this model, recognizing that each profile likely needs a unique approach necessitating a team with a wide skill set. Some studies have discussed individual patient characteristics that are suitable to outpatient virtual care, including transportation issues (eg, living far from the location of service or lack of vehicle), busy work or family schedules that make seeking in-person care difficult, and those whose conditions impair treatment-seeking (eg, anxiety, agoraphobia) [20]. The patient profiles identified from our data hone in on the archetypes that benefit most so that services can be designed and delivered with the needs of these groups in mind. Depending on priority areas, gaps in other services, or availability of resources, the model could pivot to focus more or less on certain groups [21].

In addition to limitations of the model itself, providers also identified challenges with resources and processes. Many of these were a result of the model being rapidly deployed due to the threat of COVID-19 and relatively low availability of resources, not necessarily a limitation of the vWard model itself. Staff were encountering rapid change alongside uncertainty about infection risk, leading to increased stress and potential for burnout [22,23]. Prior to COVID-19, the learning curve associated with the pivot to delivering care virtually had been documented as a common limitation to the general adoption of virtual care [3,21]. This model was resourced with minimal levels of equipment and staffing, as well as a lack of the interprofessional teams typical of hospital care. Although these resource limitations were significant, the model was sustained through dedicated staff, a shared vision, and adaptive leadership styles. As discussed by Laur et al [21], these facets are critical to managing rapid change in the health care system. Going forward, these limitations could be rectified with additional investment. There is evidence of cost-effectiveness for home-based virtual care delivery compared to in-person care [12,24]. Beyond savings to the health care system, patients also report direct savings when able to receive care at home [25]. This makes a case for greater investment in the growth of these models beyond the pandemic, alongside ongoing evaluation of impact.

Through the creation of the driver diagram, we propose a blueprint for the future vWard as an alternative model of care that leverages technology. The COVID-19 pandemic has had a negative impact on the mental health of the population due to increased stress, isolation, and reduced treatment access [26]. Additionally, in our region, we have a higher burden of mental illness in the population and a higher rate of mental health presentations to EDs compared to the rest of Canada [27]. New strategies are needed. Furthermore, our province experiences significant regional variation in access to mental health care, with disparities increasing when moving north toward rural areas [27]. Although this has not been a focus to date, virtual models of care could address geographical barriers to access with the vWard having the potential to fill a major service gap. Urgent telemental health programs have been developed in rural settings to provide assessment, with some programs offering follow-up care [28]. The vWard expands on this with the goal of reducing hospitalization that often takes individuals away from their communities and families, aiming to provide intensive care to support crisis resolution. The ability to refer to a follow-up service provides additional options for emergency mental health teams; for example, access to a telephone-based peer-led navigation service reduced the rate of admission following emergency telepsychiatry assessment to urban and suburban areas in North Carolina, United States. Although not significant, this low-resource intervention provided a signal of possible impact [29]. Drawing on the evaluation of home-based crisis resolution teams in the UK National Health Service [9], a follow-up service that includes a prescriber and well-trained multidisciplinary team members to support a range of health and social needs may be most successful at reducing rates of admission and repeat acute care use.

### Limitations

One limitation of this study is the unique circumstance under which this model was developed and launched. The catalyst for virtual care delivery and innovation provided by the pandemic was indeed an opportunity but also created conditions that do not normally exist in health care service design [30]. The survey was voluntary and thus subject to response bias; however, we achieved a very good representation of roles and range of perspectives as evidenced by the variety of staff respondent groups, which are proportional in size to the total number of individuals working in these roles. We did not collect demographic or other respondent characteristics to assess representativeness across the workforce profile. A significant gap in this work is the lack of patient perspectives, which we were unable to comprehensively collect due to resource limitations during the study period. With a plan to sustain the model locally, we are now building in patient satisfaction and further evaluation of patient experience. Other studies of outpatient virtual care have assessed patient satisfaction, generally finding high ratings, with many of the themes overlapping with those identified by our providers [4,25]. This unique blend of crisis support and virtual care requires further exploration from the patient perspective. The findings of this study must also be taken in context of its implementation: rapid, low-resource, and limited budget. Many of the “problems” of this home-based virtual model identified by the providers were instead areas that could be improved with increased investment of staff and equipment and are not necessarily intrinsic issues to the model. This is also why we focused on developing a driver diagram for a future vision based on the learnings of this rapid pivot in care delivery. These drivers and interventions will need to be validated and tested. The next step to completing the driver diagram is to map on an implementation plan along with process and outcome measures that can be collected over time, as illustrated in the Action Effect Diagram described by Reed et al [31]. Collection of these measures is crucial to evaluate the impact of the interventions.

### Conclusions

The COVID-19 pandemic advanced an opportunity to develop a novel model by leveraging technology to provide care virtually to a high-acuity population. Despite some challenges with resources in a rapidly changing health care context, we have demonstrated high levels of provider acceptance and a range of mechanisms by which the model works for a variety of patient archetypes. These findings highlight barriers to be anticipated and overcome in the design of similar models and identify the patients who may benefit most from virtual crisis intervention as an alternative to staying in a facility, be it a hospital or CSU-type environment. There is still room to improve and optimize this model. These early learnings provide a foundation for growth, sustainability, and spread going forward beyond the pandemic to increase access to quality care using novel means that are highly patient-centered. Other jurisdictions interested in developing similar initiatives may use these learnings as a starting point in the design and implementation of local programs.

## Appendix

Multimedia Appendix 1 Driver diagram for the future state of the virtual mental health crisis ward (vWard) implementation.

## Abbreviations

CRC: Crisis Response Centre
CSU: Crisis Stabilization Unit
ED: emergency department
vWard: virtual mental health crisis ward
