# Supplementary figures and images for: Early Learning From a Low-Resource COVID-Response Virtual Mental Health Crisis Ward: Mixed Methods Study
Source: JMIR Form Res. 2022 Nov 4;6(11):e39861. doi: 10.2196/39861 (PMC9640197; doi:10.2196/39861)

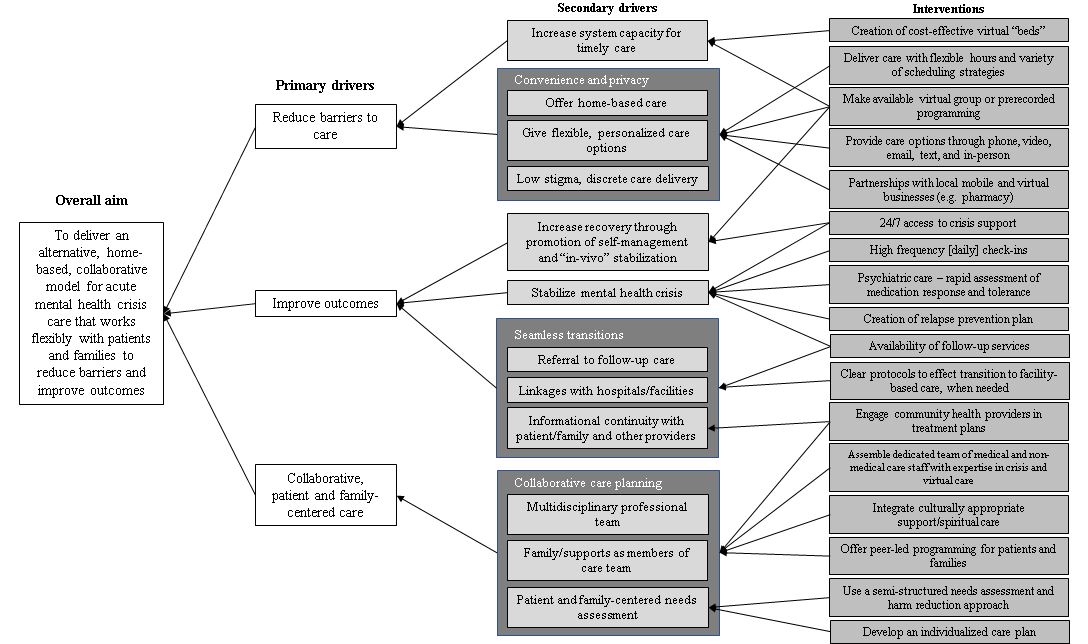

Supplement: Multimedia Appendix 1 [file formative_v6i11e39861_app1.docx]
